# Osteopontin – a biomarker of disease, but also of stage stratification of the functional myocardial contractile deficit by chronic ischaemic heart disease

**DOI:** 10.1080/14756366.2019.1587418

**Published:** 2019-03-07

**Authors:** Bogdan-Ioan Coculescu, Gheorghe Manole, Gabi Valeriu Dincă, Elena Claudia Coculescu, Cristina Berteanu, Cristina Mariana Stocheci

**Affiliations:** a Faculty of Medicine, Titu Maiorescu University, Bucharest, Romania;; b Center for Military Medical Scientific Research, Bucharest, Romania;; c Faculty of General Nursing, Bioterra University, Bucharest, Romania;; d Clinical Hospital Colentina, Bucharest, Romania;; e Faculty of Dental Medicine, Carol Davila University of Medicine and Pharmacy, Bucharest, Romania;; f Neolife Clinic, Bucharest, Romania;; g Faculty of Sciences, University of Pitesti, Pitesti, Romania

**Keywords:** Osteopontin (OPN), diastolic heart failure (DHF), preserved ejection fraction, biomarker

## Abstract

The study analyses the significance of the plasmatic values of the OPN dosed to 91 people suffering from diastolic cardiac dysfunction with preserved ejection fraction, thus revealing significant growths of its level compared to the normal value. Despite being a clinical research, its conclusions are a breakthrough, differing from the results of other studies published in the relevant medical literature. We can make this assertion because this study analyses the clinical information given by the circulating values of the OPN, based on experimental models (animals), or on patients with congestive heart failure, which can be identified with the existence of a low systolic flow. The results of our study allow us to assert that the plasmatic values of this glycoprotein lead to its acceptance in the medical practice as a new biomarker that provides indicators regarding the stratification of risk with the patients suffering from heart failure of the diastolic dysfunction type, but whose systolic flow is preserved.

## Introduction

Heart failure is one of the major public health problems where life expectancy may be prolonged by the ability of the physician to establish an early diagnosis and to intervene to prolong the duration of the evolution of each pathophysiological stage of myocardial contractile deficit. Among the factors intervening in the development of cardiovascular pathology, not only at the endothelial coronary level, but also at the level of the myocardial syncytium, is also the osteopontin (OPN). First identified in 1986, structurally, this is a phosphorylated glycoprotein produced by secretion of osteoblasts. Subsequently, it has been demonstrated that cytokine is secreted by activation of macrophages, T lymphocytes, smooth myocytes, endothelial cells, as well as and cardiomyocytes and fibroblasts^1–4^.

**Figure 1.**
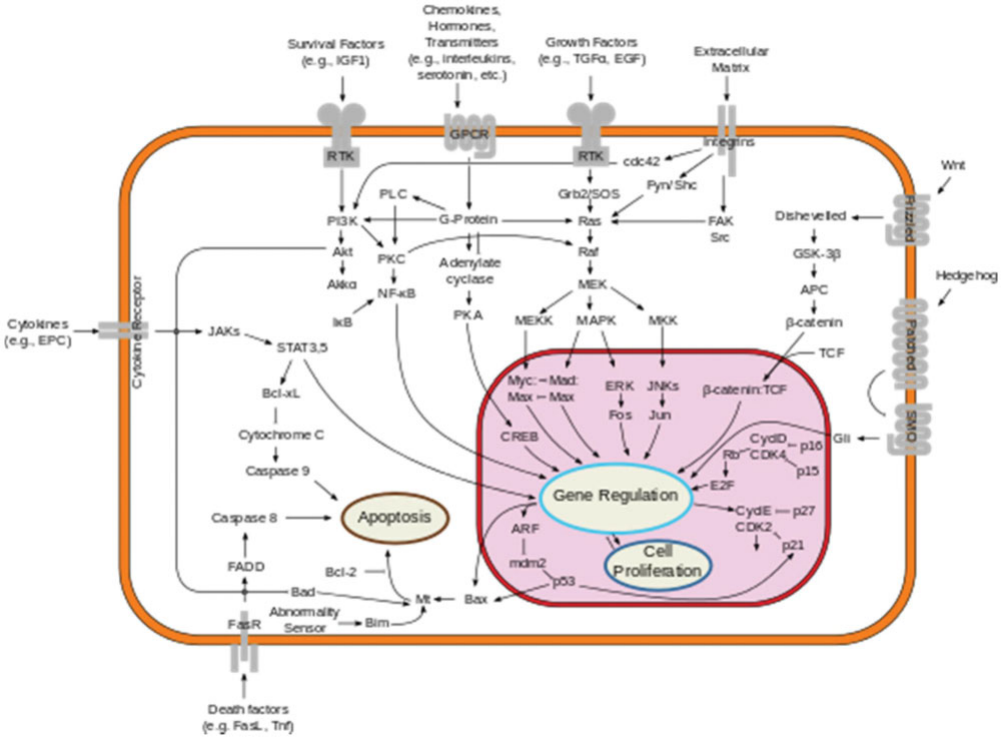
Communication between the interstitial matrix and cardiomyocytes, via activation of the integrin receptor. Activation of myocardial intra-fibrous synthesis pathways leading to myocardial remodelling and fibrosis^10^.

At myocardial level, OPN presents itself under two forms: stationary, situated in the interstitial matrix, and as a soluble cytokine, having the structure of the RGD field made up of the following amino acids: arginine–glycine–aspartate^2^^,^^4–6^. RGD mediates the inter-action with various integrins, including that with 1-integrina dominantly expressed in the myocardium. As representative of the transmembrane receptors, it provides through the soluble form of the OPN the communication between the extracellular matrix and the cardiomyocytes^5–9^ (Figure 1).

The above-mentioned activated paths allow the intervention of the matrix OPN whose synthesis is stimulated by the angiotensin II (Ang II)–aldosterone system to intervene in the development of myocardial remodelling and fibrosis^2^^,^^4^^,^^5^. Regulation at the myocardial level of these two processes is the synergistic action of OPN and Ang II, via the activation of the inflammatory process^1–4^^,^^11^^,^^12^. Besides, there are also the modulations of the apoptosis process and the facilitating of the survival of cardiomyocytes, the result of which is the installation of an inotropic function deficit^13^^,^^14^. This also contributes to the remodelling of the myocardium, which is an imperfect process, leading to an increase in myocardial contractile deficiency^11^^,^^15–18^.

## Materials and methods

The objective of the study was to establish whether there are scientifically significant statistical arguments to support the admission of serum concentration of OPN as a biomarker of cardiac failure of ischaemic etiology and preserved systolic blood flow.

From the scientific point of view, based on the existence of homeostasis of the internal environment, and also on the results of the experimental studies on animals with heart failure, it is acknowledged that the value of the tissue concentration of OPN is similar to that in the plasma, both having increased values^19^^,^^20^.

The study group consisted of 91 patients (35 women and 56 men) with chronic heart failure with myocardial ischaemia etiology.

This study was conducted in accordance with the ethical standards of the Declaration of Helsinki.

**Figure 2.**
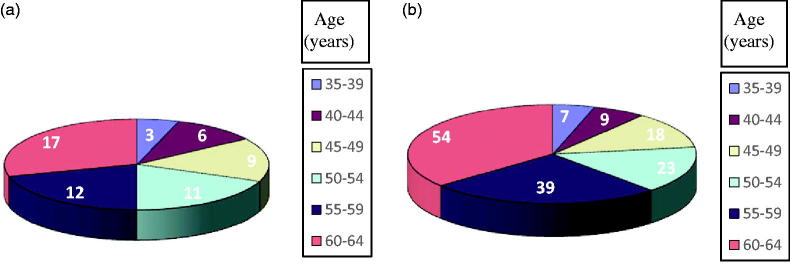
Distribution of the study cases in relation to patients’ sex and age group.

Although the literature does not record the influence of the age on the circulating concentration of OPN, we mention that in the studied group the average age was 54.55 years: 53.86 years for women and 55 for men (Figure 2).

The criteria for selecting the subjects for the study group can be divided into: inclusion and respectively exclusion criteria.

### Inclusion criteria

The inclusion criteria consisted of the clinical, laboratory, and paraclinical elements which allow the establishment of cardiac failure clinical diagnosis and its class on one hand, and on the other hand the etiologic diagnosis of chronic ischaemic heart disease (IHD), but without differentiation between the acute and the sequelae myocardial infarction.

1. The etiological diagnosis of myocardial contractile deficit, chronic IHD is anamnestically supported by the presence of stable angina pectoris of effort and the results of paraclinical explorations.
  Thus, in order to evaluate the functional status of the ventricular myocardium in diastole, all patients underwent:
  - heart – pulmonary radiography;
  - determination of venous pressure;
  - electrocardiogram of rest, and in cases where its appearance did not reveal the existence of ischaemic changes, the stress test was used.
  - echocardiography (bidimensional evaluation, M-mod). Regarding the practical utility of this exploration, the literature notes that it is the best noninvasive method of assessing the diastolic function and appreciating the filling pressure of the left ventricle (LV)^11^^,^^21^.
  - oximetry.
2. The left ventricular myocardial contractile deficit was evaluated according to the NYHA classification (Table 1).
  Prior to inclusion in the study, patients signed an informed consent for the use of clinical data, accepting the full set of investigations required for conducting the intended scientific process and analysis of paraclinical and/or serum determinations’ results for the purpose of the research.

**Table 1.** Classification of heart failure (NYHA criteria).

Class	Clinical/severity of symptomatology
I	Asymptomatic; absence of physical activity limitation.
II	Absence of symptoms at rest; occurrence of symptoms in conditions of normal, as duration and intensity, physical activity.
III	Absence of symptoms at rest; symptomatology present in conditions of physical activity, with characteristics below those that define a normal one.
IV	Symptoms present at rest.

### Exclusion criteria

There were no co-ops in the batch, patients diagnosed with the investigated type of disease that had received medication over the course of the last 3 months: statins, immunoglobulins or phosphodiesterase inhibitors.

We justify this attitude through the following actions of medication:

- For hypolipidaemic agents:
  - the development of pleiotropic effects by improving endothelial function as a result of constitutive nitric oxide synthase (cNOS) gene transcription induction^22^;
  - minimising the production of reactive oxygen species (ROS) in the arterial wall^21–23^;
  - the very likely reduction of macrophage production of tumour necrosis factor-alpha (TNFα), interleukin-1 (IL-1), and interleukin-6 (IL-6)^11^^,^^24^^,^^25^.
- Exclusion from the study group of patients who had received phosphodiesterase inhibitors over the last 3 months was required because they inhibit the cytokines production, especially the one of IL-6, IL-1β, and TNFα with a role in ventricular myocardium inflammation and remodelling processes.
- The non-acceptance of congestive heart failure (CHF) cases that had received immunoglobulin for the same 3-month period was justified by the reasoning of a possible interference between this type of pharmaceutical agents and the inflammatory process which develops in the DHF^26^^,^^27^.

Another incompatibility of not being included in the study group was the association with chronic inflammatory diseases such as lupus erythematosus, rheumatoid arthritis, ankylosing spondylitis, infectious endocarditis, psoriasis, because it could have influenced the results of serum determinations due to the inflammatory action of autoimmune type^28^.

Method used for serum OPN dosing: plasma OPN was measured by enzyme-linked immunosorbent assay (ELISA) using recombinant human OPN ELISA. Normal values: 0.312–20 ng/mL.

## Results

**Figure 3.**
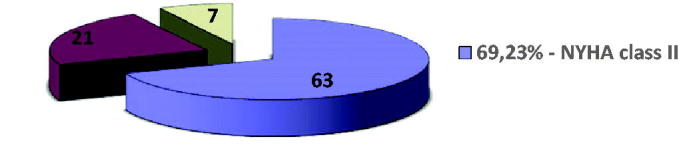
Distribution of batch case size by classes of contractile deficit, using the NYHA classification criteria.

The classes of contractile functional decompensation of the heart were those of the NYHA classification, the patients included in the batch framing in grades II–IV as follows (Figure 3).

**Figure 4.**
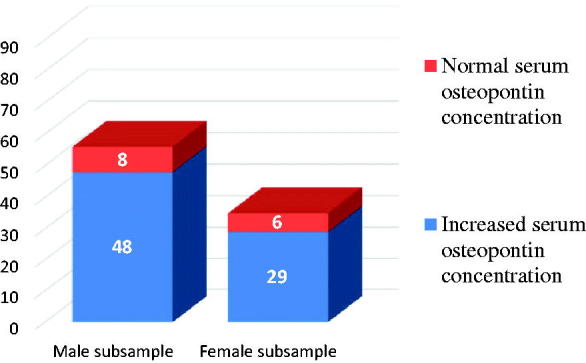
Comparison of the incidence of serum values of OPN in the batch, depending on the sex of the patients.

In the study group, the distribution of the cases with normal and increased serum values among each same-sex subgroup revealed that only eight male and six female patients presented physiological OPN serum concentration (Figure 4).

The study of Figure 4 shows that elevated serum OPN values are present in the batch at a rate of 78% (77 cases), 3.5 times higher than normal (14 cases = 22%). The incidence of increases may be considered as approximately identical among both the male and the female subsample, although there is a slightly difference of 2.5% in favour of the male gender:

$$\frac{\text{Men with elevated serum OPN values}}{\text{Women with elevated serum OPN values }}=\frac{48\text{ cases }}{29\;\mathrm{cases}}=\frac{85.5\%}{83\%}$$

The comparison between the mean serum concentration and standard deviation values of OPN in patients with diastolic dysfunction with those of circulating OPN levels normally admitted in healthy subjects reveals a net increase, even if the ejection fraction is not affected (Figure 4). Thus, the increase in mean serum OPN is 2.5 times higher than the upper limit of normal circulating:

$$\frac{\text{Average OPN serum concentration in the study sample }}{\text{Normally accepted circulating OPN levels}}=\frac{50.7\pm 21.7\;\mathrm{ng}/\mathrm{mL}}{<20\;\mathrm{ng}/\mathrm{mL}}$$

**Figure 5.**
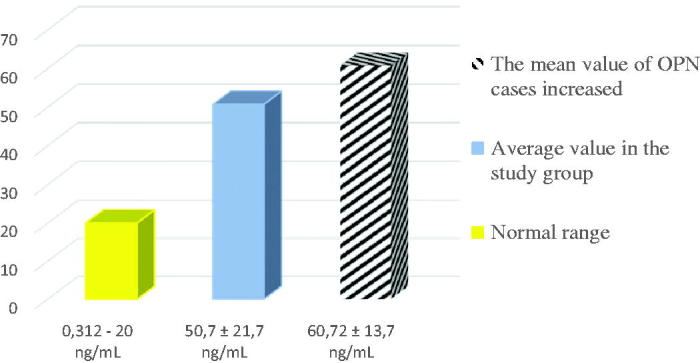
Comparison between the normal OPN serum values and the medium serum OPN concentration of the research sample.

The medium serum OPN concentration was calculated for the entire sample based on the individual results for the biomarker, also including the patients with normal values, as presented in the following figure (Figure 5).

Subsequently, for each class of myocardial contractile deficit, serum OPN and standard deviation were calculated. The calculation also included the normal OPN values present in some cases (Table 2).

**Table 2.** Serum mean values and standard deviation of OPN in relation to the myocardial contractile deficit class.

Class functional	Incidence in batch		The calculated mean value of the domain (ng/mL)
	No. absent	%	
II	63	69.23	45 ± 18
III	21	23.08	51 ± 20
IV	7	7.69	56 ± 27
Total/media	91	100	50.7 ± 21.7

The determined values allowed us to determine the incidence of the average serum concentration of OPN for each class of myocardial contractile deficit as outlined in Table 2.

The mean serum concentrations and the standard OPN deviations depending on the patients’ sex for each CHF class are shown in Table 3.

**Table 3.** The mean serum concentrations of OPN in relation to the sexually co-opted patient.

CHF class	Calculated mean value of the domain (ng/mL)		
	Whole lot	Men sublot	Women sublot
II	45 ± 18	49 ± 20	41 ± 16
III	51 ± 20	53 ± 21	49 ± 19
IV	56 ± 27	58 ± 29	54 ± 25
Total/media	50.7 ± 21.7	53.3 ± 23.3	48 ± 20

## Discussions

The choice of chronic IHD as an etiology responsible for myocardial inotropic deficiency may be motivated by the fact that hypoxic tissue state exacerbates the production of ROS/RNS. The consequence is the installation of oxidative stress, a state in which ROS induces DNA degradation, or small changes in certain domains of various functional protein molecules, interactions that inhibit them or cause premature apoptosis of cardiomyocytes through caspase and/or kinases cascade activation^11^^,^^21^^,^^29^ (Table 4).

**Table 4.** The main protein intracellular targets influenced by ROS/RNS^30^.

Structural type of the intra-cardiomyocyte target	Main intercellular targets	
Protein macromolecules from a certain cascade complex	Cytokines	
	Growth factors	
	Hormones	
Protein molecules	Protein kinases (A, G, C), jun kinases (JNKs), protein kinase B (Akt)	
	Rho protein family (Ras)	
	Transcription factors	
	Protein activator 1 (AP1) Nuclear factor (NF)-kB p53 protein	
	Metabolic enzymes	

For the patients studied, in the case of patients with normal serum values of OPN, the results were:

$$\frac{\text{Male cases with normal serum OPN values}}{\text{Female cases with normal serum OPN values }}=\frac{8\text{ cases }}{6\;\mathrm{cases}}=\frac{14.5\%}{17\%}$$

The percentage difference of 2.5% in the incidence of normal serum levels of OPN between the two sexes is not statistically significant. Thus, there are no significant differences with respect to the sex of the patients, between the incidence of both the elevated and the normal values, as a preliminary conclusion can be reported that sex is not a determinant of the circulating level of OPN.

The incidence of high levels of OPN serum in men, 1.7 times compared to that in women, allows us to admit and claim that the processes of myocardial reshuffle (hypertrophy and fibrosis) among the patients with DHF by IHD are more frequent and more intense in men than women (Table 3).

The comparison between the high levels of OPN serum taking into consideration the average age of the patients with DHF caused by chronic IHD reveals an earlier increase in female patients. This result of the study may be considered as an argument in support of the hypothesis that the degree of OPN activity is regulated to a certain extent hormonally.

Also, in the batch patients, we evaluated whether high OPN levels offer independent prognostic information about diastolic dysfunction. We have found the existence of a correlation between the plasma concentrations of this biomarker and the class of heart failure. Thus, the mean serum concentration of OPN increases by 1.3 in patients with grade IV cardiac failure, compared to the same parameter existing in Class II NYHA cases of cardiac failure (Table 2). In support of our findings, there comes the research provided by Rosenberg et al. whose results demonstrated that in heart failure patients without or with minor dysfunction symptoms, the average serum concentration of OPN was lower than the one found in patients with moderate or severe disease, where the OPN levels were almost triple^5^.

We appreciate that the particular mode of variation of OPN serum concentrations, in direct correlation with the severity of heart failure symptomatology, recommends the determination of serum OPN concentrations not only as a biomarker of heart failure but also as a staging/stratification biomarker of the contractile functional deficit induced by myocardial hypoxia. We also subscribe to studies that recognise that circulating OPN levels can be taken as a biomarker and a prognosis factor in heart failure^31–39^.

Before concluding, although not a result of our study, but considering that it is a useful tool for medical practice, we are inserting the results of the last decade research that argue that by measuring the circulating level of OPN, information can be obtained on how the patients would respond to anti-aldosterone diuretic therapy^3^^,^^36^^,^^40^. Thus, the dynamic tracking of serum concentration of OPN is also useful as a biomarker for evaluating the effectiveness of therapy. According to Maisel et al., the value of OPN as a biomarker in heart failure can be increased by integrating it as a multimarker^41^ (“multimarkers strategy” – 11,21).

## Conclusions

The results of the study lead to the following conclusions:

1. The value of the OPN circulating level is influenced by gender, regardless of whether or not there is a manifest disease. We consider such a conclusion to be useful because it assumes that OPN's response, as an activity, is determined both by hormones, beyond the possibility of direct, para- or metacrine stimulation. It is necessary for this scientific supposition to be researched more thoroughly by studies on a statistically significant casuistry, especially since the literature has already introduced the possibility of OPN concentration measurement into the current medical practice, without referring to its conditionality and field of application.
2. Based on the existence of a higher incidence of elevated OPN serum levels in the whole lot, but also in men, compared to women, we claim that the myocardial remodelling processes (hypertrophy and fibrosis) are more frequent and more intense in males with DHF.
3. The correlation between the severity of symptomatology in DHF and the circulatory level of OPN recommends that serum concentrations be determined not only as a cardiac failure biomarker or as one of stratification of contractile deficiency, but also as a prognostic marker of this affection.
